# Considerations on the Failure Mechanisms at Fatigue Loading of 1018 Steel Samples Coated with Wip-C1 by Cold Spray

**DOI:** 10.3390/ma17081868

**Published:** 2024-04-18

**Authors:** Layth Alkisswani, Viorel Goanță, Corneliu Munteanu, Fayez Samara, Roxana Elena Cosau, Bogdan Istrate

**Affiliations:** 1 Mechanical Engineering, Mechatronics and Robotics Department, Mechanical Engineering Faculty, Gheorghe Asachi Technical University of Iasi, 700050 Iasi, Romania; layth.alkisswani@student.tuiasi.ro (L.A.); fayez.samara@student.tuiasi.ro (F.S.); elena-roxana.cosau@student.tuiasi.ro (R.E.C.); bogdan.istrate@academic.tuiasi.ro (B.I.); 2 Technical Sciences Academy of Romania, 26 Dacia Blvd., 030167 Bucharest, Romania

**Keywords:** interface damage, coating, cold spray, fatigue, crack initiation, SEM analysis

## Abstract

There are some important advantages presented by metal specimens coated with WIP-C1 (Ni/CrC)-type materials. However, given the coating methods and the stress under dynamic loads, there are issues that need to be taken into account, particularly in terms of the behavior at the interface between the two materials. Using standardized cylindrical 1018 steel specimens uniformly coated with WIP-C1 (Ni/CrC) by cold spraying, this study investigated the fatigue behavior of the specimen as a whole, focusing on the interface areas of the two materials. The fatigue life diagram is given, to a large extent, by the behavior of the base material. As a result, in this work, we have focused not so much on the fatigue behavior of the assembly as on the integrity of the coating material and the defects, failures, etc., that may occur at the interface after a certain number of cycles. The applied load was cyclic fatigue through alternating–symmetric cycles. Scanning optical microscopy was used to observe plastic deformations and crack propagation during the breakage process. It was found that both the base material zone and the cover material zone presented good performance when the maximum stresses were at low values. A fatigue durability curve was also plotted, showing a conventional appearance for a metallic material, slightly influenced by the destruction of the base material interface. At higher maximum stress and, consequently, to large strains, a series of destructions at the interface of the two materials, of different types, were observed and will be highlighted in the paper.

## 1. Introduction

AISI 1018 is a low-alloy carbon steel with a high manganese content (0.6–0.9%), which gives it good mechanical machinability. This manganese content also gives it a certain brittleness (though still low), observable in the final breakage section, without posing problems under fatigue stress. Deformation at low stress values is minimal, resulting in good dimensional stability with a specific elongation of only 15% (l0 = 50 mm). AISI 1018 steel welds very well and can be used in the production of casings, structures for overhead bridges, cranes, armors, etc. An important feature, relative to its accessible cost price, is its high Brinell and Rockwell C hardness values (126HB and 131HRC) [1]. This steel, being suitable for certain applications, raises the issue of improving some characteristics through coating [2]. Various coating methods are used in accordance with laboratory capabilities and the base material’s ability to support both the coating material and the preservation of intrinsic properties as a result of the coating process. With the coating, there is protection of the surface from the surrounding environment: high temperatures, corrosion, erosion, action of chemical agents, etc. In general, scientific research papers present studies on the influence of different coating parameters on the quality of the resulting coating material [3,4,5,6,7] rather than on analyzing the mechanical properties of the assembly resulting after coating [8,9,10,11,12,13,14,15]. Furthermore, the study of fatigue behavior at the coupling interface of the two materials is found in a few research works.

In the investigation of the paper [16], a 6082 aluminum alloy coated by cold spray method with powder of the same material was analyzed, this being prepared by gas atomization in an argon atmosphere. The results obtained and presented were micrographs of the interface area, residual stresses, and fatigue behavior. The depth variation of the residual stresses is presented, quite briefly, with the specification that the maximum stress is compressive and is about (−200 MPa). As far as fatigue tests are concerned, only the fatigue strengths for the different types of samples used are presented. The number of specimens used for each series is not given, nor is the way in which that value of fatigue strength was assigned to each of the sample series tested. Using a sample type described in ASTM-B93 (2009), Ghelichi et al. [17] made judgments on fatigue behavior (R = −1) by providing S-N (strength–number of cycles) plots on cold spray-coated aluminum alloy samples under different conditions and making comparisons with the uncoated sample. In the paper [18], Bagherifard s.a. conducted a review of the fatigue behaviour of components fabricated by cold spray (CS) deposition, presenting a number of remarkable results such as fatigue properties of CS deposits, fatigue crack initiation, and growth behaviour, the effect of pre-surface preparation or post treatments on fatigue performance, comparing cold spray with other deposition technologies in terms of fatigue behaviour.

Fatigue endurance is a phenomenon that begins with the initial degradation of the material, necessitating a study of the possibility of crack initiation and propagation, which, in the case of coating methods, is most likely to occur at the interface. Base materials used for coating by the cold spray method include pure elements, some Al and Ti alloys, composite materials, stainless steels, and Inconel-type superalloys. The deposition process involves accelerating powder particles through a Laval nozzle using He or N_2_ as preheating gases, with particle velocity in the range of 300–1200 m/s. The speed and dimensions of the coating particles influence the aspect of the surface and subsurface layers of the base material, forming a bond zone with the base material at these locations. After the intermediate layer is formed, the coating process is continued using particle-to-particle bonding of the coating powder. Cold spraying (CS) is a high-performance and high-productivity technique used to add additional properties to components to make them applicable in different fields of interest [19]. The materials used in the Cold spray process can be composite materials [3,4], some aluminum or titanium alloys [5], stainless steels [6], Inconel 718 type superalloys [8] or pure elements [20]. In this deposition process, metal–ceramic powder particles are accelerated to high speeds in the range of 300–1200 m/s by a Laval nozzle using preheated gases (He or N_2_). The particles strike the surface and form a bonded layer on the surface of the base material.

By bonding the already-formed particles with the newcomer particles at speed/temperature, the coating is carried out layer by layer until the desired thickness of the coating area is achieved [21]. Due to the high ductility of Ni, increased deposition efficiency reported to the other parameters used was obtained for Cr_3_C_2_-NiCr coatings using the following parameters: spray distance, 18 cm; air flow, 600 L/m; air pressure, 6 kg/cm^2^; oxygen flow, 250 L/m; and oxygen pressure, 9 kg/cm^2^ [22]. Ni and NiCr are known to have good corrosion and oxidation resistance at high temperatures, with similar coefficients of thermal expansion. This minimizes the effect of residual stresses that could arise due to different thermal expansions [9,10].

There are research studies presenting the cold spray coating process while highlighting its high efficiency. On the other hand, H. Singh et al. highlight a number of influences on the behavior of the component that has undergone deposition by the cold spray method [23]. Influence parameters such as low pressure or high pressure, characteristics of the two materials coming into contact, particle velocity, splat adhesion, the effect of particle diameter, the nature of carrier gas, the effect of temperature, the effect of oxidation condition, the effect of Nozzle Design, and the effect on microstructure are analyzed.

When using the low-pressure system, it is considered that operational safety is improved and the cost of spraying is significantly reduced compared to an HPCS system, but the deposition efficiency with this system usually does not exceed 50%.

In the high-temperature sputtering system, the solid particles are accelerated and hit the surface of the solid medium with high energy, leading to complete mechanical and/or metallurgical bonding. The efficiency of this method is calculated to be about 90%.

Particle velocity is an important factor in cold spraying in relation to the nature of the materials used. For a given material, a minimum addition particle velocity, known as the critical velocity, must be ensured. Only particles that reach a velocity higher than the critical velocity ensure the formation of a coating.

Powder particles, depending also on their nature, can deform plastically on impact with the substrate surface, and, once stuck to the surface, are called splat. The interlocking of these particles forms the coating. There is a difference between the diameter of the initial particle and the maximum cross-sectional size of the particle after adhesion. Obviously, the mechanical characteristics of this particle also change, as it undergoes differential plastic deformation in relation to the differential velocities. Therefore, coatings will be smoother at high impact velocities, increasing with increasing gas temperature. Dickinson et al. [24] observed that increasing the pressure from 0.4 MPa to 1 MPa of cold-sprayed TiO_2_ particles on a stainless steel substrate will also increase the adhesion of splat particles. The results show that smaller splats (<5 μm) had higher adhesion strength than larger splats (>5 μm).

It is found that there is an interdependence between the diameter of the coating particles and their velocity, and thus: Vp = k/dn, where Vp is the particle velocity, and k and n are coefficients related to the driving gas conditions for a given material [15]. Hence, the effect of particle velocity on the most accurate formation of the adhesion layer should be reviewed.

In the CS method, the most used gases are nitrogen and helium. The latter is the best solution because it is inert and allows the highest particle velocity to be achieved. Li et al. [25] reported that the particles had a higher velocity when helium was used compared to nitrogen as the driving gas. However, helium is 10 times more expensive than nitrogen, making it economically unviable for many applications unless recycled. In some applications, a mixture of nitrogen and helium is used as a carrier gas. Nitrogen (N_2_), being a diatomic gas, with its addition in He, increases the enthalpy of the carrier gas providing better heat transfer with the spray particles, but also reduces the velocity due to the heavier atomic mass, leading to coatings with reduced density and hardness [26,27].

In scientific papers, it has been reported that particle velocity increases with increasing temperature. Although preheating the coating particles leads to higher velocity, it also increases the risk of oxidation or nitriding, as the case may be, which can be detrimental to the bonds between particles [28].

In recent times, improvements in nozzle shape and size have resulted in higher deposition velocities of the coating mixture and, consequently, higher deposition capacities. This leads to higher-density coatings, and therefore higher deposition efficiency. Nozzle measurements that influence particle velocity are inlet diameter, throat diameter, outlet diameter and expansion ratio (ratio of outlet to throat area), inlet convergent section length (upstream length), and outlet divergent length (downstream length).

The microstructure of the cold coating is affected by particle velocity. The surface of the base material is heavily impacted by the first deposited particles, while the outer state of the coating is more porous. The thickness of this porous layer is influenced by the properties of the coating particles and the morphology of the deposited particles. The effect of tailing is more effective when using helium [29], which resulted in a thinner top layer than with nitrogen.

Taking all these considerations into account, the aim of the present work is to study and make some considerations on the fatigue behaviour of AIS 1018 steel coated by cold spray method with WIP-C1. In this study, the morphology of crack initiation and propagation is analyzed, both at the interface between the two materials and in their volume, after the specimens were subjected to stress fatigue until failure by alternating–symmetric cycling.

## 2. Materials and Methods

### 2.1. Coating Process

Standardized specimens according to ASTM E8/E8M-16 Tensile Testing of Metallic Materials [30] were made of AISI 1018 alloy steel. All specimens were cleaned, after which, using a VRC Gen-III cold spray machine (VRC Metal Systems, LLC., SD, USA), they were circumferentially coated with a WIP-C1 powder, as the name is used commercially. The main characteristics used in the deposition process are shown in Table 1. In order to achieve good adhesion of the coating material to the base material, a thin layer of WIP-BC1 powder with adhesive characteristics was applied to the surface using a nozzle orientation of 600. In addition to creating a thin layer of WIP-BC1-type adhesive coating, this operation also achieves the following: cleaning and stress relieving the surface of the base material and obtaining a certain roughness beneficial for the subsequent bonding with WIP-C1. Uniform deposition on the cylindrical surface of the specimen was achieved by rotating the specimen between the bars of a lathe and moving the nozzle longitudinally/axially while keeping it perpendicular to the specimen axis. The parameters used in the actual spraying process were those indicated in Table 1 and are identical to those used in previous research [15,31].

The X-ray diffractions (XRD, Panalytical, Almelo, the Netherlands) were performed using an Xpert PRO MPD 3060 facility from Panalytical (Almelo, the Netherlands), with a Cu X-ray tube (Kα = 1.54051 Å), 2 theta of 10°–90°, step size of 0.13, time/step of 51 s, and a scan speed of 0.065651°/s.

**Figure 1:**
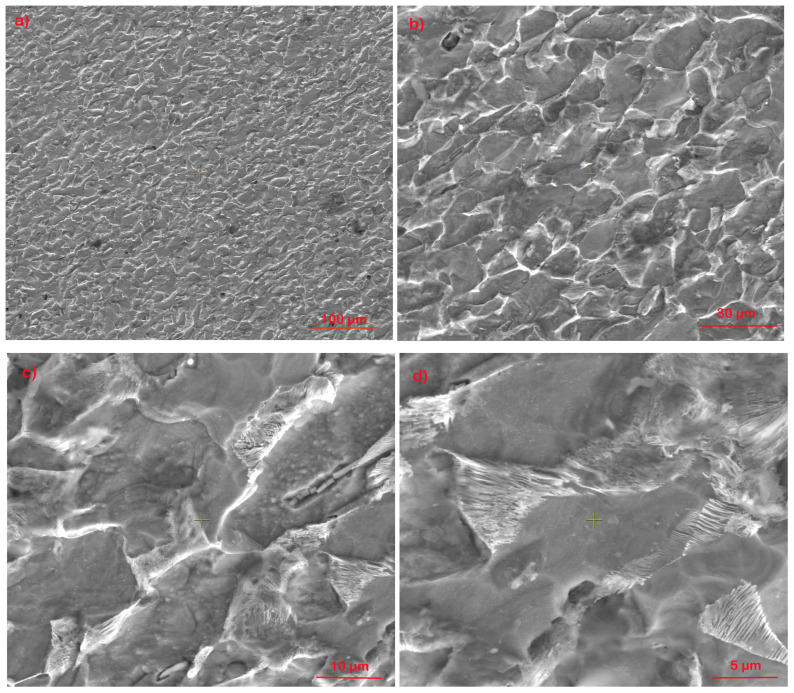
Electron microscopy images of the base material, 1018 steel, at different magnifications: (**a**) 500×; (**b**) 1000×; (**c**) 5000×; (**d**) 10,000×.

Figure 1 presents the metallographic structure of the substrate, which is specific to low-alloy steel composed of equiaxial ferrite grains and lamellar perlite grains.

**Figure 2:**
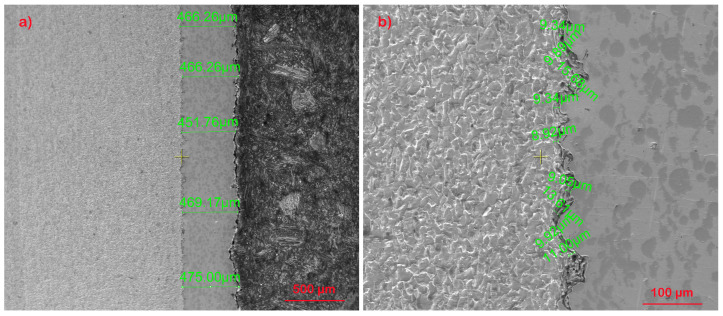

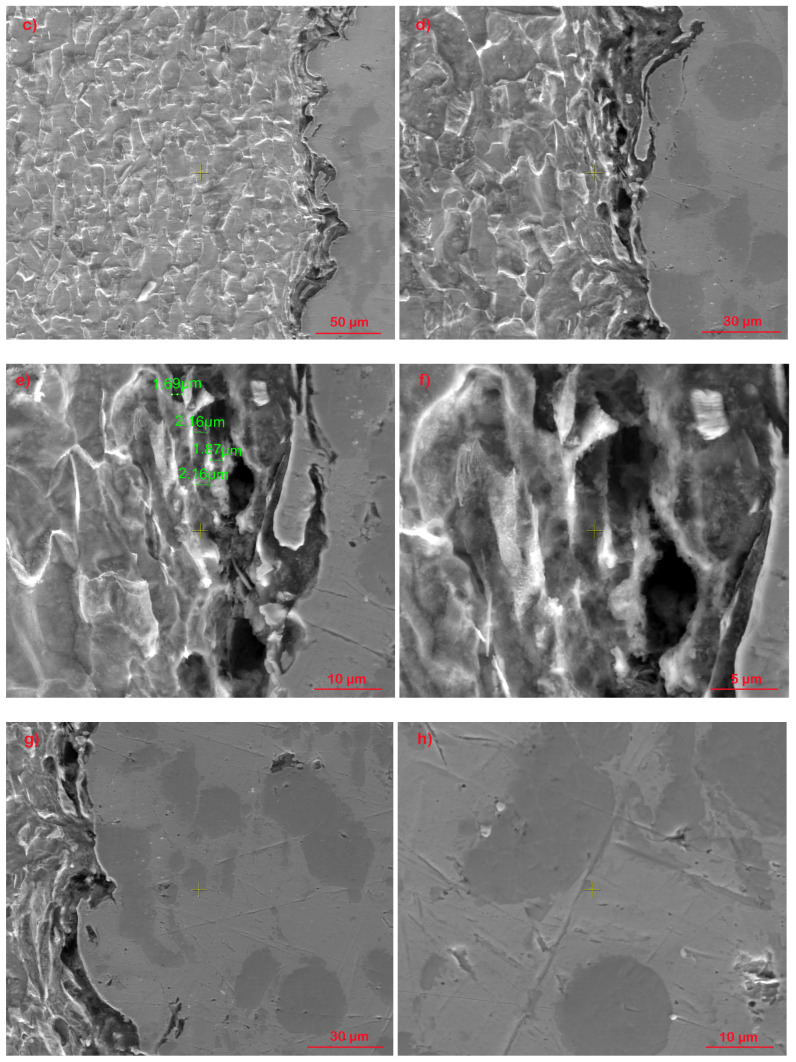
Cross-section images of the interface between the base material and the coating at different magnifications: (**a**) 100×; (**b**) 500×; (**c**) 1000×; (**d**) 2000×; (**e**) 5000×; (**f**) 10,000×; (**g**) 2000×; (**h**) 5000×.

In Figure 2, the thickness of the layer obtained by the “cold spray” method is highlighted, having an average thickness of 465 µm (a). When analyzing the coating–substrate interface, a non-uniform profile specific to this type of deposition is observed, which is based on the mechanical interlocking at very high speeds of the metallic particles. Thus, in the base material, local plastic deformation is highlighted on a thickness of approximately 10 µm (b), where, at high magnification powers, an elongation of the ferrite grains is observed in the impact area with particles sprayed at supersonic speeds (c–e). In Figure 2f, partially deformed areas of the perlite structure are observed, while in Figure 2g,h, a structural homogeneity of the deposition layer and the presence of hard, undeformed particles embedded in the Ni-based matrix are highlighted.

**Figure 3:**
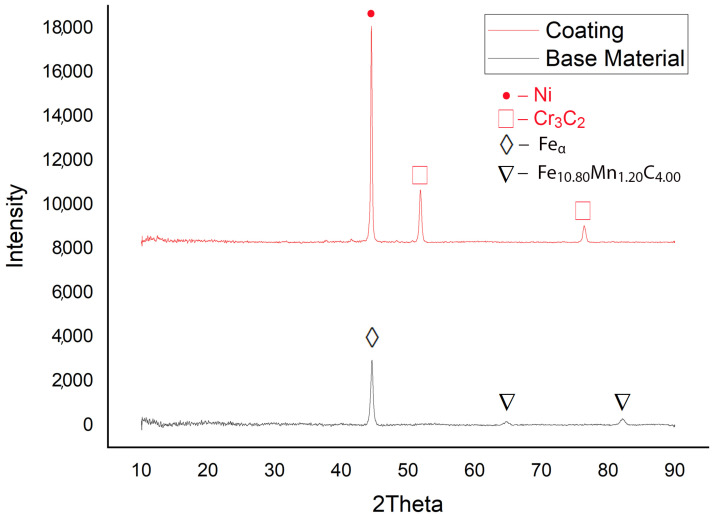
Comparative diffractograms for the base material–coating.

In Figure 3, the comparative diffractogram of the base material–coating structure is highlighted. Thus, the presence of predominant phases of Ni in the deposited layer having a cubic crystallographic structure (C.O.D. Ref. Code: 96-210-0647), and of Feα in the base material having a cubic crystallographic structure (C.O.D. Ref. Code: 96-901-3473) is observed. In the diffractograms, the presence of chromium carbides (Cr_3_C_2_) hard particles highlighted in Figure 2g,h as being the secondary phases in the coating layer, having an orthorhombic crystallographic structure (C.O.D. Ref. Code: 96-591-0109) was observed. In the base material, the secondary phase is formed from the compound Fe 10.8Mn1.2C4 with an orthorhombic crystalline structure (C.O.D. Ref. Code: 96-901-6678).

However, at the interface, penetrations of the coating material into the base material can be seen up to 10 μm deep. The way of penetration and surface damage of the base material differs from sample to sample. Under these conditions, given that the specimens will be subjected to fatigue and that any non-uniformity may lead to crack propagation, it will be found that at close stresses the specimens will fail after a different number of cycles.

### 2.2. Tensile Testing and Fracture Analysis at Static Loading

**Figure 4:**
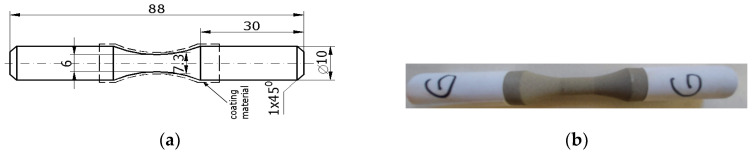
The tensile fatigue test sample: (**a**) shape and dimensions of the sample (mm), (**b**) aspect of the coated sample.

The aim of this work was to, after fatigue loading at alternating–symmetric cycles of cold spray-coated cylindrical specimens, study the behavior both at the interface between the base material and the additive material and in the volume of the two materials. It is known that, currently, parts that are to work under dynamic stresses can be designed in different zones of the stress–number of cycles (σ-N) durability curve. Consequently, in this work, we plotted the durability curve (σ-N), making observations on the behavior under cyclic fatigue stresses on the broken specimens for each stress level. To plot the curve (σ-N), it is necessary to start from high stress levels but below the yield limit of the material. In this sense, preliminary static tensile tests were performed to determine both the yield limit and the general deformation characteristics of the material subjected to coating. In this context, it was considered to determine the predominantly ductile or predominantly brittle character of the material which helps us in setting the fatigue test parameters. The static tensile characteristics of the material are determined in accordance with the ASTM E8/E8M-16 standard [30]. Moreover, static tensile tests were performed on both a coated sample and an uncoated sample, both to highlight certain differences and to observe the stress and specific deformation at which cracks can appear in the coating layer, through visualizing the outer surface. The tested samples had the shape and dimensions presented in Figure 4a,b.

**Figure 5:**
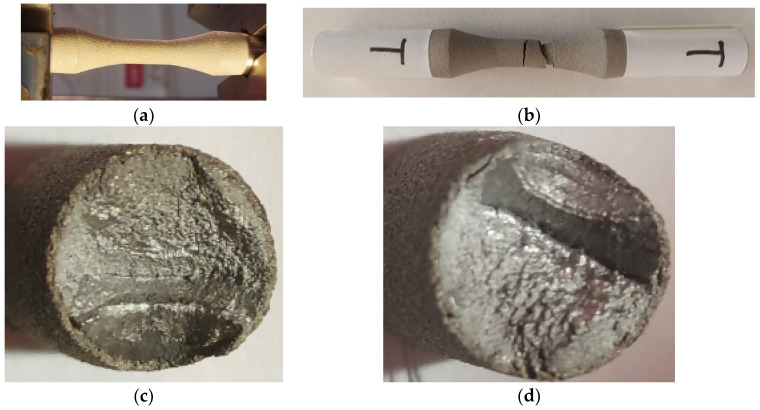
Sample T—static tensile test: (**a**) view of the specimen during tensile fatigue testing; (**b**) sample breakage mode—base and coating materials; (**c**,**d**) the surfaces resulting from the breakage at tensile fatigue.

**Figure 6:**
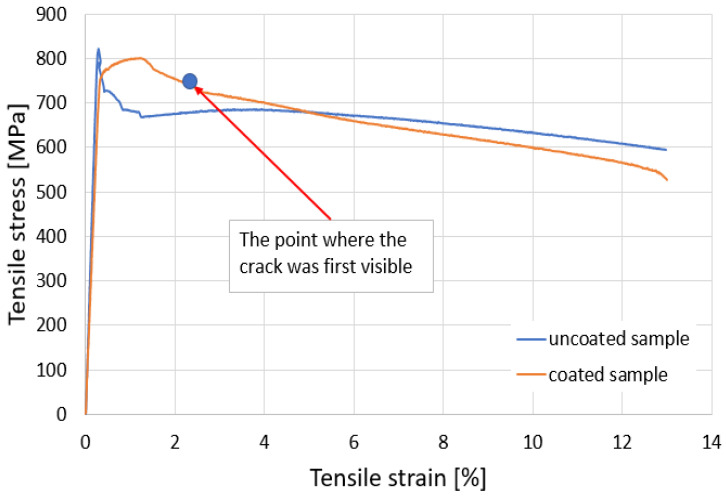
Stress–strain variation, coated and uncoated samples.

In Figure 5, images from the static tensile test of the coated sample are presented. Figure 5a shows a view of the sample during stress, Figure 5b shows the mode of breaking of the outer surface that includes the coating material, and Figure 5c,d presents the breakage in the cross-section of the sample. From the analysis of the specific stress–strain curve, shown in Figure 6, and from observing the surfaces that resulted from breaking, some appreciations can be made and some conclusions can be drawn:

(i) The initial elasticity zone is large, reaching high values of stress on a linear portion;
(ii) No strain hardening zone of the material is observed in the characteristic curve; after reaching the offset yield point Rp_0.2_ (σp_0.2_), the material undergoes pronounced elongation, but without any further increase in force;
(iii) The appearance of the curve is mainly influenced by the behavior of the base material. However, it can be noted that, due to the surface of the base material being bombarded with the coating particles, changes appear in the shape of the stress–strain curve specific to the coated sample. The maximum stress is lower for the coated sample, but the yield zone presents higher stresses;
(iv) The total elongations of the two samples are similar, at approximately 13%, with a decrease in breaking stress for the coated sample;
(v) After the appearance of the first crack in the coating material, it suffers significant damage because the values of the elastic constants of the two materials differ substantially;
(vi) The surfaces resulting from static stress breaking, for the base material, are approximately perpendicular to the direction of stress, which shows the slightly brittle character of this material;
(vii) The coating material presents two breaking zones. These are determined by the appearance of the first crack in the coating material and the fracture of the base material in another zone, which also determines the breaking of the coating material;
(viii) The yield limit (σp_0.2_) was determined to be 726 MPa and was taken from the data table corresponding to the static tensile test.

### 2.3. Fatigue Analysis Performed on Coated Samples

**Figure 7:**
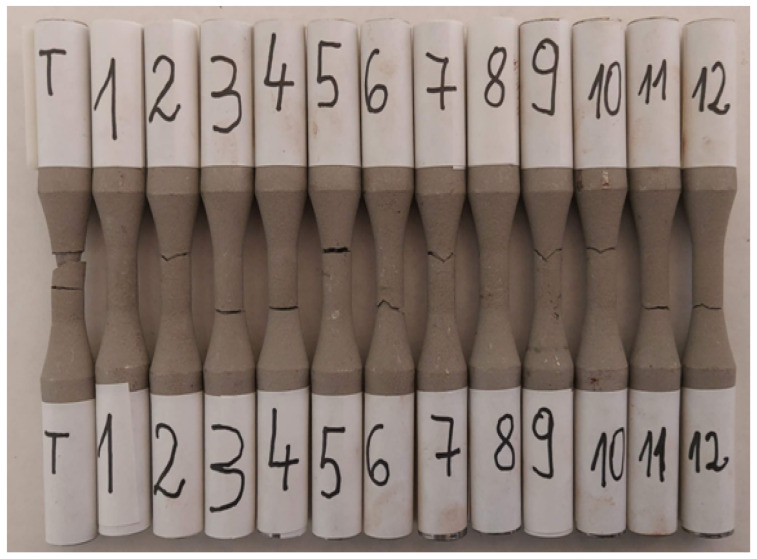
Samples after fatigue testing.

The fatigue stress was applied to the 12 AISI-1018 steel samples coated with WP-C1, shown in Figure 7, with sample T being subjected to static tensile testing.

The tests were conducted considering the provisions of the ASTM E466-15 standard [32]. The shape, dimensions, and appearance of the coated samples tested for fatigue are presented in Figure 4a,b. The stress was applied on an Instron 8801 machine following a symmetric alternating tensile cycle with the first stress value of 601 MPa so that the stress remained in the elastic domain considering that the yield limit was 726 MPa (σ_max_/σ_y_ = 0.82). A reference value of 5 million for the number of cycles N_0_ was chosen. If, after this number of cycles, the sample did not break, the testing was stopped. In this context, samples 1 (σ = 435 MPa) and 8 (σ = 424 MPa) have had no failures before reaching 5 million cycles. The maximum stress level and the number of cycles are shown in Table 2.

**Figure 8:**
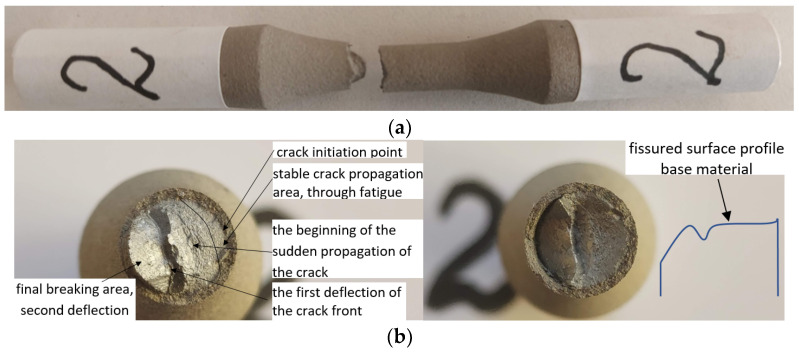

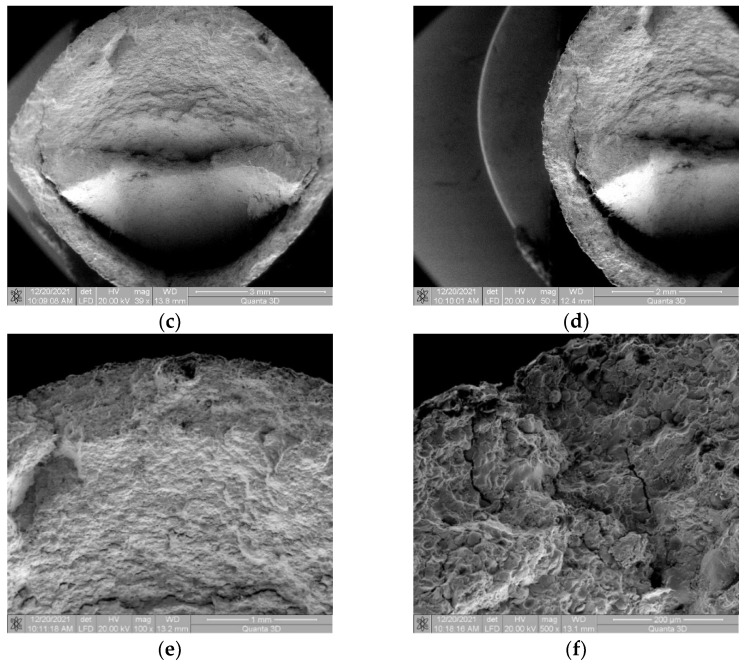
Macroscopic and microscopic appearance of broken surfaces for sample 2—σ = 601 MPa, N = 12,141 cycles.

The representation of figures for each sample tested until failure includes:

- A macroscopic side view of the broken sample after fatigue stress (Figure 8a);
- A macroscopic view of both ends resulting from the break (Figure 8b) and a front view, taken immediately after stress.

Using the Quanta 3D SEM microscope, four photographs were taken and added to the macroscopic ones for identification and observation of breakage parameters. These included:

- An overview image, with a magnification of approx. 30×, to be compared with the photo taken with the electronic microscope (Figure 8c);
- An image of the final, abrupt break area, (Figure 8d);
- A close-up image in the crack initiation region with a magnification of 50× (Figure 8e);
- An image with a magnification of 500× from the crack nucleation/initiation area highlighting the initial crack (Figure 8f).

The two materials in contact and in adhesion exhibit in adjacent areas different levels of deformation given that ε_base_ > ε_coating_. Within the fracture surfaces a strong failure of the coating material was observed due to the different deformation modes at the interface of the two materials. Large deformations generally occur at high values of stress introduced into the specimen as a result of the fatigue loading.

## 3. Results and Discussion

This research on fatigue behavior included 12 samples subjected to symmetric axial-cyclic traction. Of the 12 specimens available for fatigue testing, before 5 million stress cycles at maximum stresses in descending order, only 8 were broken. Observations on a few samples with more significant characteristics are presented as follows. Samples that were stressed at close stress values and exhibit the same breakage aspects are analyzed together in the paper, as figures, presenting only the specifications for a single sample.

### 3.1. Sample 2 (σ_max_ (MPa); N), (601; 12141)

#### 3.1.1. Macroscopic Observations

Stable crack propagation through fatigue initially occurs perpendicular to the direction of stress followed by two successive deflections of the cracked surface, as shown in Figure 8a–c. For sample 2, due to the relatively high stress introduced, the initial stable crack propagation surface is small, as shown in Figure 8b. The beginning of unstable propagation also occurs perpendicular to the direction of stress, as shown in Figure 8c. In this sample, as in some of the following samples, a first deflection of the crack front is observed during its abrupt propagation. Subsequently, the crack front changes its propagation direction. This occurs as an effect of massive dislocation movement under high stress in the remaining unbroken area, on a different plane than the initial crack front propagation. Hence, the crack front’s direction is at approximately 45°.

#### 3.1.2. Microscopic Observations

In the final area of the fatigue breaking surface, the detachment of the coating material from the base material can be seen. This refers to the crack that appeared between the base material and the additive material, not to the detachment from the area of the crack front’s deflection at a 45° angle within the base material. This detachment is explained by the large deformations of the base material in the final break area compared to the smaller deformations of the coating material, as shown in Figure 8c,d. In the crack initiation area, shown in Figure 8e,f, cracks in the coating material and detachments of material at the interface with the base material are observed. When the crack propagates, in its immediate vicinity, plastic deformations occur in the base material that are much larger than the deformations in the cover material. This is due to the fact that the base material has a certain ductility with relatively large plastic deformations (approx. 15%) while the cover material is a predominantly brittle material with much smaller, mainly elastic, deformations. The damage in the coating material is significant because the stress level is relatively high, at approx. 67% of the ultimate tensile strength (σ_uts_ = 897 MPa). Through the coating material, both circumferential and radial cracks are observed, as well as intergranular and intragranular cracks.

### 3.2. Sample 3 (σ_max_ (MPa); N), (530; 38234)

#### 3.2.1. Macroscopic Observations

**Figure 9:**
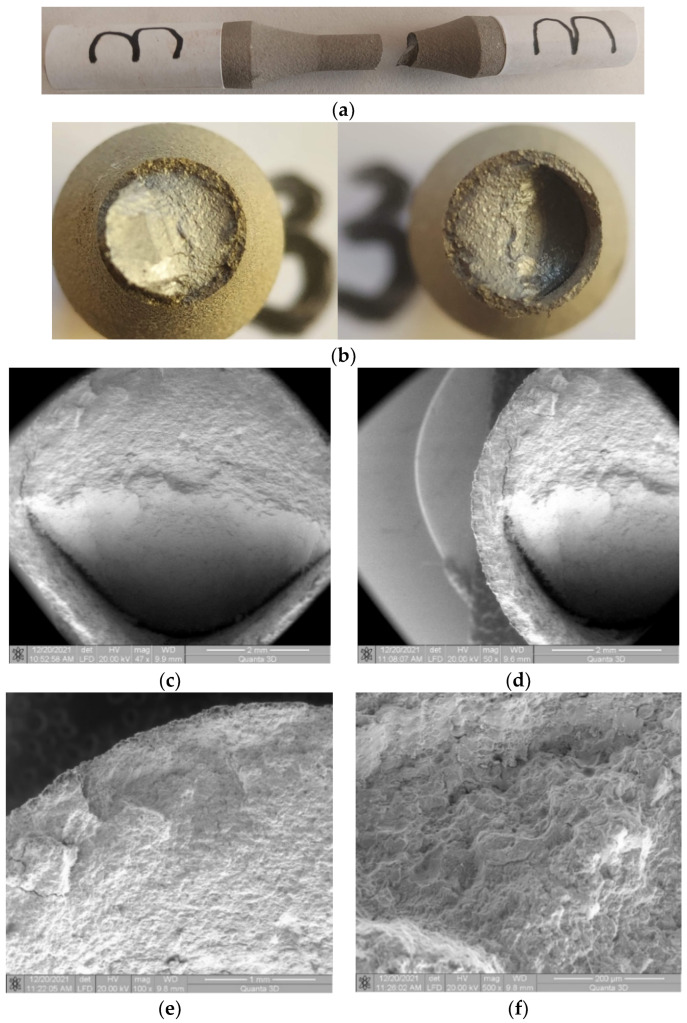
Fractures in specimen 3, macroscopic and microscopic views—σ = 530 MPa, N = 38,234 cycles.

This sample also shows the deflection of the cracked surface. Unlike the double deflection, the area approximately perpendicular to the direction of stress is smaller, as shown in Figure 9a–c. It is noted that the stress level is approx. 60% of the ultimate tensile strength. The blockage in the path of controlled crack propagation being the normal stress (surface perpendicular to the direction of stress) is large, leading to early deflection of the crack.

#### 3.2.2. Microscopic Observations

It is noted that in the area of deflection of the crack front, a detachment (crack) appears between the base material and the coating material, as shown in Figure 9d. Moreover, the surface of the coating material remains perpendicular to the direction of stress, with crack propagation controlled by normal stress, while the base material propagates at an angle, propagation controlled by tangential, shear stress. This difference between the two modes of propagation leads to the respective detachment. In the crack initiation area, Figure 9e,f, significant damage to the coating material is again observed: material detachments, and a crack between the coating material and the base material. However, compared to the previous sample, no such large cracks are observed in the coating material area.

### 3.3. Sample 4 (σ_max_ (MPa); N), (495; 54704)

#### 3.3.1. Macroscopic Observations

**Figure 10:**
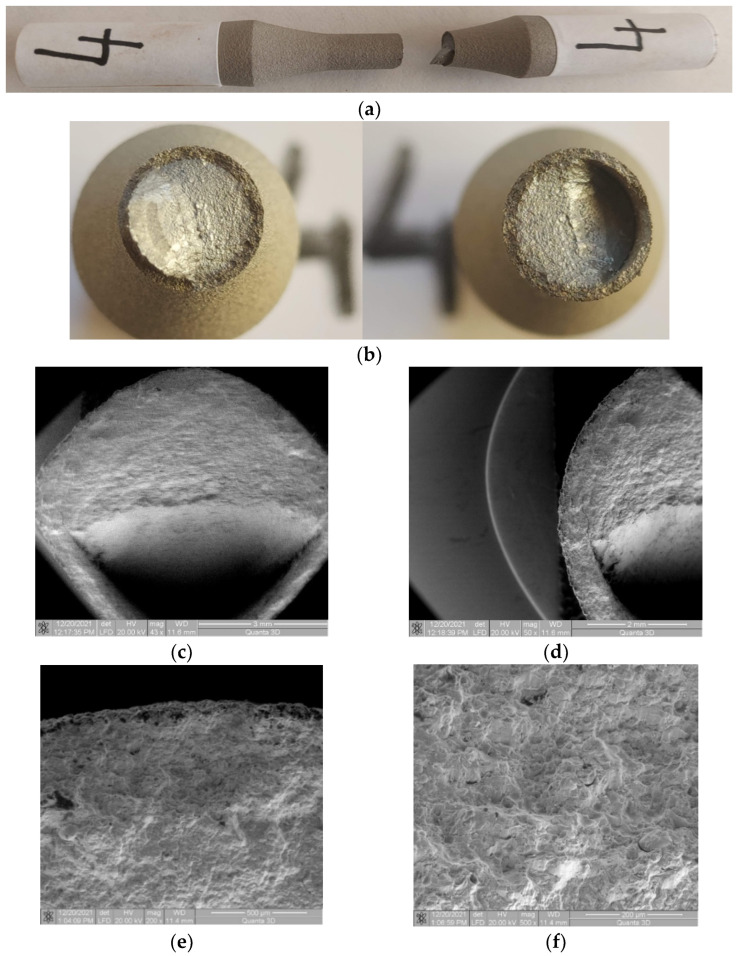
Fractures in specimen 4, macroscopic and microscopic views—σ = 495 MPa, N = 54,704 cycles.

From a macroscopic perspective, the breaking of sample 4 is similar to that of sample 3. The difference lies in the larger area of propagation perpendicular to the direction of stress. Consequently, the area with deflection is smaller than in the previous sample, as shown in Figure 10a–c. And the area of stable crack propagation is larger, explainable by the lower stress level. Obviously, the double deflection does not occur, with the initial crack propagation area much closer to being flat.

#### 3.3.2. Microscopic Observations

In this sample, it is observed that, in the area of entry with the deflection of the crack front, detachments of the coating material in relation to the base material occur on both sides of the deflection. The cracks are smaller in length compared to those presented in the previous sample, here the stress level is also lower, as shown in Figure 10c,d. In the crack initiation area, shown in Figure 10e,f, no significant damage to the coating material is observed. The existing cracks are of small length and are local, caused by the different directions of the crack front’s propagation in relation to the direction of the formation of the composite structure of the coating material.

### 3.4. Samples 5, 6, and 7 (σ_max_ (MPa); N), (459; 74787), (452; 88823), (449; 100970)

It is specified that the stresses were so close because it was not known from the beginning what the maximum stress value was for which the samples would not break after five million cycles. As seen from Table 2 (where results were placed after all tests were completed) at a maximum stress of 435 MPa, the sample did not break before five million cycles.

#### 3.4.1. Macroscopic Observations

**Figure 11:**
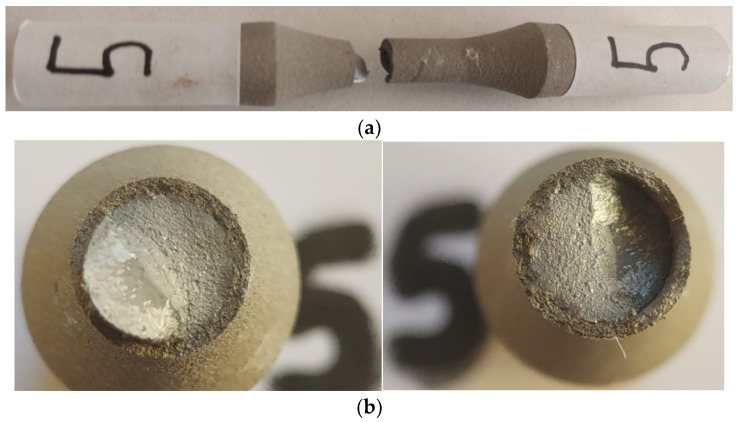

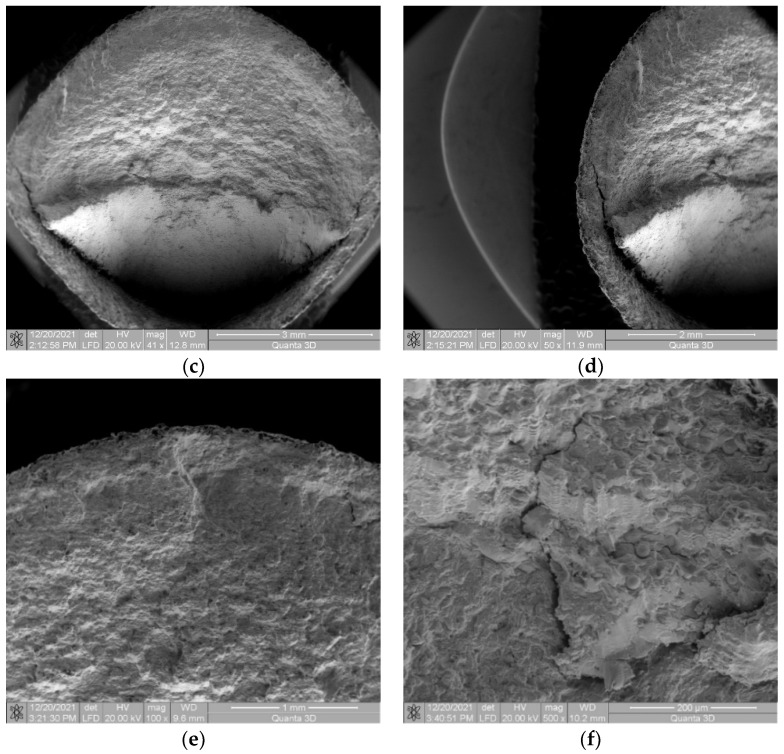
Fractures in specimen 5, macroscopic and microscopic views—σ = 459 MPa, N = 74,787 cycles.

From a macroscopic perspective, the breaking of samples 5, 6, and 7 is similar to each other. The flat, perpendicular area to the direction of stress is larger and distinct and the stress level is lower, as shown in Figure 11a–c. Here, the area of stable crack propagation is larger, being close to the area where the deflection of the crack plane occurs. It is found that, when the strength reserve of the cracked surface is at its lower limit due to stable crack propagation, sudden and unstable propagation starts directly by the inclination of the propagated crack relative to the direction of loading. This does not happen in samples 2 and 3 where the sudden crack propagation started earlier.

#### 3.4.2. Microscopic Observations

From Figure 11d,e, it is observed that large cracks appeared at the interface of the coating material with the base material in the area of the beginning of the crack direction deflection. The crack starts with the detachment of the coating material from the base material and continues in the coating material, being mechanically weaker. In contrast, in the crack initiation area, Figure 11e,f, no significant damage to the coating material is observed. The crack in the coating material in the area of fatigue crack initiation propagated being controlled by normal stress and propagation through the base material. A small crack (detachment) is observed between the base material and the coating material.

### 3.5. Samples 9, 10, 11, and 12 (σ_max_ (MPa); N), (445; 131110), (442; 234291), (440; 461533), (438; 738335)

#### 3.5.1. Macroscopic Observations

**Figure 12:**
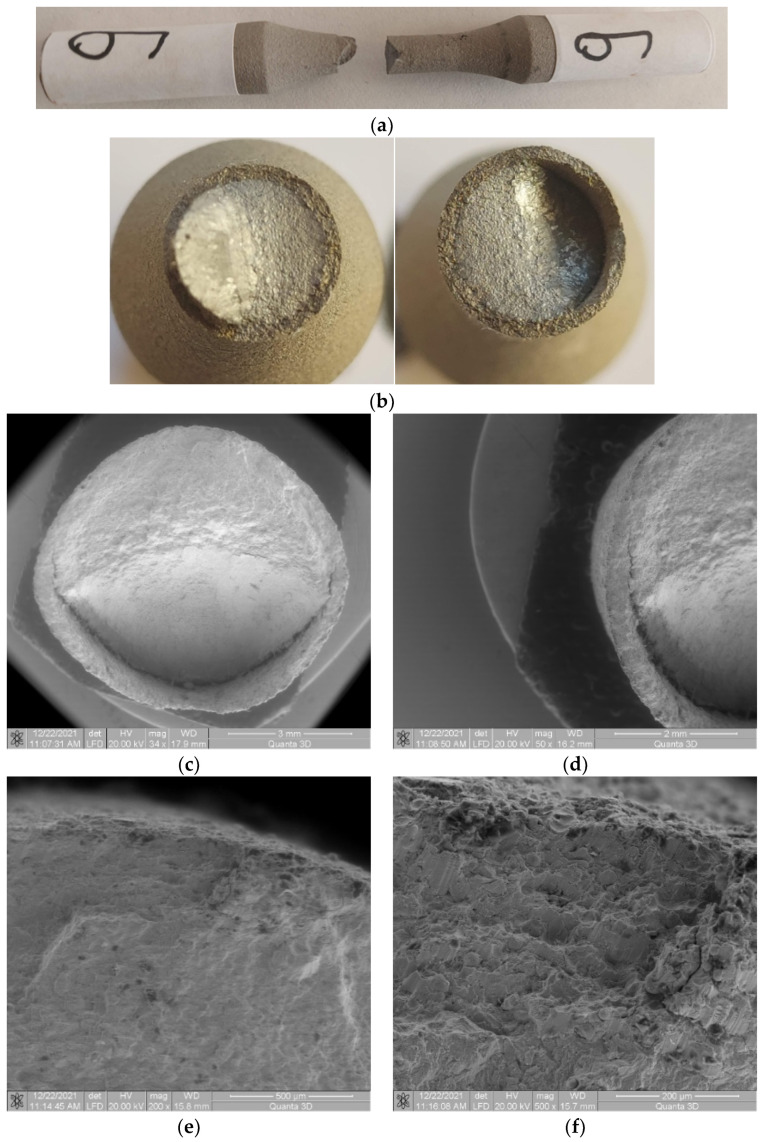
Fractures in specimen 9, macroscopically and microscopically views—σ = 445 MPa, N = 131,110 cycles.

No significant differences in breaking between samples 9, 10, 11, and 12 are observed, with the stresses being very close, at 445 MPa, 442 MPa, 440 MPa, and 438 MPa for sample 9 and 449 MPa for sample 7. Remember that sample 1, being stressed at 435 MPa, withstood more than five million cycles. Thus, for the tests of samples 9, 10, 11, and 12, we had a test range of only 14 MPa. Under these conditions, differences in the testing stress are small, as is the breaking behavior, both of the base material and the coating material. In this sample, the deflection of the cracked surface is also observed, as shown in Figure 12a–c, when the sudden crack starts to propagate. At a lower stress level, the area that is straight and perpendicular to the direction of stress is larger and more distinguishable, as shown in Figure 12a–c. Here, the area of stable crack propagation is larger, being close to the area where the deflection of the crack plane occurs. The area of abrupt crack propagation begins in the immediate vicinity of the crack deflection area. In Figure 12b, the very well-outlined cylindrical surface of the cracked surface belonging to the coating material, from the area of stable fatigue crack propagation, can be seen.

#### 3.5.2. Microscopic Observations

Here, too, cracks appear between the coating material and the base material in the two opposite directions of the deflection of the propagated crack front, as shown in Figure 12c,d. It is also noted that in the area of stable crack propagation through fatigue, the appearance of the cracked surface in the coating material is normal, without observable damage and without deflections of the cracked surface. Unlike the area of stable propagation, in the area of abrupt propagation, distortions are observed in the appearance of the fatigue-broken surface in the coating material. In the crack initiation area, Figure 12e,f, no detachment of the coating material from the base material through the appearance of cracks is observed. From Figure 12f, it can be seen that the breaking of the structure in the area of the coating material occurred intragranularly—see the appearance of the broken surface in the area of the coating material. Hence, the conclusion is that, at low stress values, the bonding forces between particles of the composite coating material are large enough to allow intragranular passage of the crack front. The radial crack observable in Figure 12f is due to a small detachment of the coating material, usually in the case of the crack initiation area.

### 3.6. Samples 1 and 8 (σ_max_ (MPa); N), (435; 5234605), (424; 5039737)

#### Macroscopic Observations

**Figure 13:**
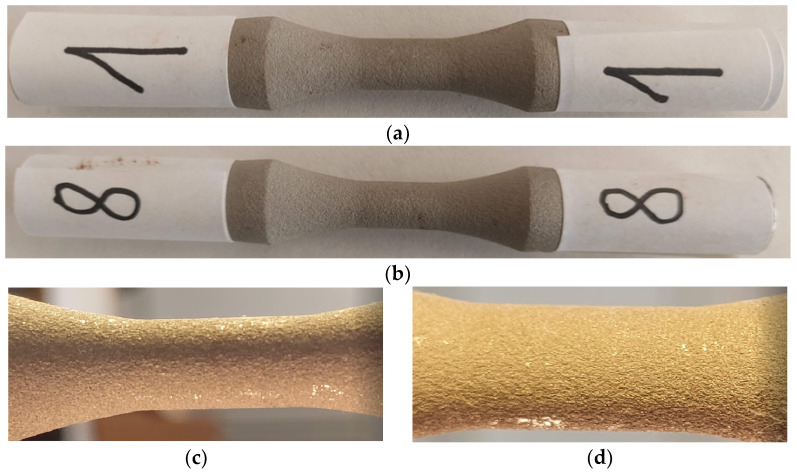
Macroscopic appearance of the surface after fatigue stress—sample 1 (**a**) and sample 8 (**b**). σ_1_ = 435 MPa, N_1_ = 5,234,605 cycles; σ_8_ = 424 MPa, N_8_ = 5,039,737 cycles; (**c**) macroscopic view of sample 1 (10×); (**d**) macroscopic view of sample 1 (40×).

Considering the experience with the fatigue behavior of previous materials, sample 1, as shown in Figure 13a, was subjected to a stress of 435 MPa, hoping for a break after a certain number of cycles that would provide an indication of lower breaking stresses. It did not break even after 5,234,605 cycles of symmetric axial alternating stress. Sample 8, shown in Figure 13b, did not break even after 5,039,737 cycles. Consequently, for the following samples compared to this one, the stress level was increased. From the macroscopic study of the external surfaces, as shown in Figure 13c,d, no deterioration (material tear-offs, microcracks, detachments, etc.) of the coating surface was observed. Therefore, it can be stated that at a symmetric alternating fatigue stress of approx. 48% of the ultimate tensile strength, the coating material performed very well. The minor damages observable in Figure 13d are due to contact during the handling of the sample for testing.

### 3.7. Wöhler Diagram for 1018 Coated Samples Fatigue Tested

**Figure 14:**
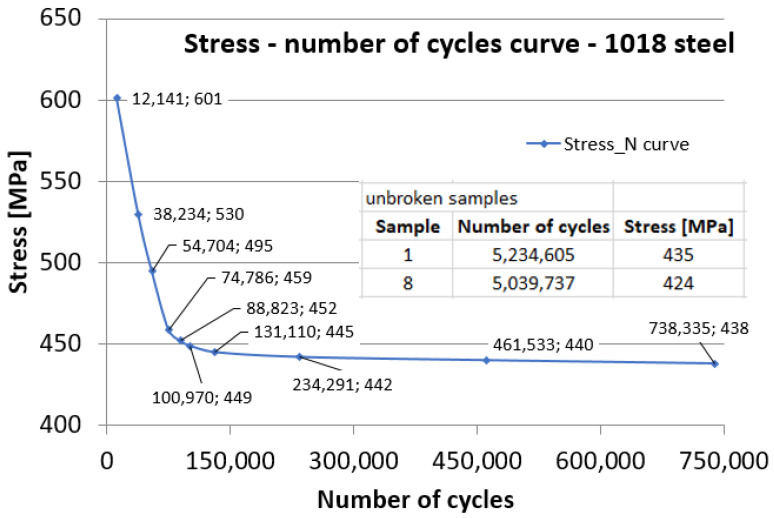
Stress–number of cycles variation for 1018 steel coated with WIP-C1 material.

Using the pairs of data, cyclic fatigue stress–number of cycles to failure, the stress–number of cycles (Wöhler diagram) was plotted, as shown in Figure 14. Out of the 12 fatigue specimens, samples 1 (435 MPa) and 8 (424 MPa) did not fail and did not show any damage, even when loading after more than five million cycles. The appearance of the diagram is conventional for a metallic material, with a fatigue limit estimated at around 430 MPa. As seen in Figure 14, it was not possible to achieve a break in samples that could withstand more than 740,000 cycles. Therefore, at very close stresses, the samples either broke at low numbers of cycles of less than 740,000 or withstood more than 5 million cycles.

## 4. Conclusions

In this research, the following characteristics and directions were determined: (i) the fatigue behavior of the assembly formed by the base material and the coating material; and (ii) the stress zone in which the coated parts can be used under fatigue stress without causing damage, either to the base material or to the coating material. When the external fatigue loading introduces stresses very close to the yield strength, major damage to the coating at the interface with the base material can be distinguished. These defects consisted of the detachment of the coating material, as well as microcracks that appeared near the crack initiation zone.

At high values of the stresses introduced by the fatigue loading, the final fracture zone is oriented at a certain angle to the loading direction. In the early region of this deformation, where deformations (especially plastic ones) are large, the coating material detaches from the base material. On the other hand, at high stresses, the area of stable crack propagation in the base material has a non-uniform appearance.

According to previous observations, for the assembly formed by AISI-1018 steel and WIP-C1 (Ni/CrC) coated material, it is not recommended to use similar components subjected to fatigue stresses over 445 MPa. For use at medium durability, coated components can be used at fatigue stresses of up to 440 MPa, at which the deterioration of the coating material is reduced. Parts made of AISI 1018 alloy steel and coated with WIP-C1 (Ni/CrC) material by cold spray method can be in operation for a long period of time if the applied fatigue stresses do not exceed 430 MPa, even in the presence of stress concentrators. At stresses below this value, no damage was observed in the tested samples. Moreover, no deterioration of the external surface of the coating material was observed on the surface of samples tested over five million cycles.

## Figures and Tables

**Table 1:** Characteristics of the fatigue specimens prepared by cold spray deposition.

Characteristic	Value
Gas	Nitrogen
Pressure	6.2 MPa (900 psi)
Temperature	675 °C
Nozzle ID	WC NZL0060
Nozzle throat size	2 mm
Powder feeder speed	10 rpm
Powder feeder gas flow	105 slm
Standoff distance	25 mm
Spray angle	90 deg.
Nozzle traverse speed	250 mm/s
Nozzle step distance	0.25 mm
Layer thickness	0.127 mm
Target coating thickness	0.508 mm
Powder	WIP-C1
Bond coat	WIP-BC1 and 60°

**Table 2:** The maximum stress (σ_max_) and the number of cycles up to failure (N), obtained for 1018 steel samples coated with Ni/CrC.

Sample No.	σ_max_ (MPa)	N
1	435	5,234,605
2	601	12,141
3	530	38,234
4	495	54,704
5	459	74,787
6	452	88,823
7	449	100,970
8	424	5,039,737
9	445	131,110
10	442	234,291
11	440	461,533
12	438	738,335

## Data Availability

Data is contained within the article.
